# Epidemiology of Inflammatory Bowel Diseases in Nepal

**DOI:** 10.7759/cureus.16692

**Published:** 2021-07-28

**Authors:** Mukesh S Paudel, Ajit Khanal, Barun Shrestha, Bibek Purbey, Bidhan N Paudel, Gaurav Shrestha, Jiwan Thapa, Khus R Dewan, Ram Gurung, Neeraj Joshi

**Affiliations:** 1 Gastroenterology, National Academy of Medical Sciences, Kathmandu, NPL; 2 Gastroenterology, Chitwan Medical College, Bharatpur, NPL; 3 Gastroenterology, Medicare Hospital Limited, Kathmandu, NPL; 4 Gastroenterology, Annapurna Gastrocare Hospital, Nepalgunj, NPL; 5 Gastroenterology, College of Medical Sciences, Bharatpur, NPL; 6 Gastroenterology, Kathmandu University Hospital, Dhulikhel, NPL; 7 Gastroenterology, Nidan Hospital, Kathmandu, NPL

**Keywords:** ulcerative colıtıs, crohn's disease, inflammatory bowel disease, epidemiology, rural nepal

## Abstract

Introduction

Inflammatory bowel diseases (IBD) comprise ulcerative colitis (UC) and Crohn's disease (CD). These are diseases of the gastrointestinal tract without a clear etiology but have strong relationships with underlying factors like genetic susceptibility, environmental factors, and intestinal bacteria. In the east, inflammatory bowel diseases predominantly affect the younger population and have an almost equal gender distribution. With urbanization and the adoption of the western lifestyle, the incidence and prevalence of IBD are increasing in Asia. In this study, we describe the epidemiology of IBD in Nepal.

Methods

This was an observational study conducted in nine endoscopy centers within Nepal. Two years of data of colonoscopies in these centers were collected retrospectively. IBD was diagnosed by endoscopic examination. The incidence of IBD was calculated as the number of patients with IBD per 1000 colonoscopies per year. The demographic profiles of the patients were also collected.

Results

A total of 7526 colonoscopies were done in nine centers within the two years study period. IBD was seen in 479 patients (6.3%). The incidence of UC was 23.7 per 1000 colonoscopies per year and the incidence of CD was 1.6 per 1000 colonoscopies per year. UC (87%) was more common than CD (13%). Both UC and CD were mostly seen in the 30 to 40 years age group. In patients with UC, the rectum was the most commonly affected site.

Discussion

IBD in Nepal affects young males in their thirties. Younger age of affliction with a chronic disease and lack of awareness regarding the symptoms and diagnostic modalities of IBD may result in a delayed diagnosis. The target population must be made aware of the presenting symptoms of IBD and a need for colonoscopic examination for diagnosis. There is also a need for creating a national IBD registry for Nepal.

## Introduction

Inflammatory bowel diseases (IBD) are defined as systemic, autoimmune, relapsing-remitting chronic diseases of the gastrointestinal system [1]. The etiology of IBD is unclear. Genetic susceptibility, environmental factors, and intestinal bacteria are implicated to be involved in the pathophysiology of IBD [2]. Inflammatory bowel diseases are generally divided into two types: ulcerative colitis (UC) and Crohn's disease (CD). In some patients, there may be overlapping pathological features of UC and CD. This condition is sometimes known as indeterminate colitis (IC) [3]. Depending on the location of lesions, the diagnosis can be suspected on colonoscopic examination and can be confirmed by further tests like radiological examination and histopathological examination.

The four epidemiological stages of IBD are emergence, acceleration in incidence, compounding prevalence, and prevalence equilibrium. In 2020, developing countries are in the emergence stage [4]. Although previously rare, there has been a two to three-fold increase in the incidence of IBD in several countries of Asia [5]. In the west, UC and CD appear to be equally distributed between both genders. However, a review published in 2012 showed that various countries of Asia have an equal gender distribution of UC but a male predominance for CD [6]. CD appears to develop at a younger age in our part of the world, whereas UC has a similar distribution as to the west [7].

In Nepal, UC is more prevalent than CD. Few single-center studies have looked into the clinical characteristics of patients with IBD in Nepal [8-11]. We aim to collect data from various centers throughout the country to assess the rate of identification of IBD by colonoscopy per year and to describe the gender and age distribution of IBD in Nepal. We have also reviewed the available literature on IBD in Nepal.

## Materials and methods

This retrospective study was carried out in various centers of Nepal. Gastroenterologists from all over the country were invited to take part in the study via electronic as well as personal communications. The endoscopists collected retrospective data from the electronic record section from their endoscopy suites. Duplicate entries were excluded. Data regarding the colonoscopies were collected from February 15, 2017, to February 14, 2019 (two years duration) and entered in a proforma.

All patients who were 14 years of age and above and who underwent colonoscopic evaluation by practicing gastroenterologists within the prespecified time duration were included in the study. IBD was diagnosed by clinical and colonoscopic features. Patients who underwent sigmoidoscopy only or those with incomplete colonoscopic examination were excluded from the study. Information regarding the age, sex, colonoscopic diagnosis, and location of lesions were collected. The extent of colonic involvement with UC was noted. Entries with missing demographic information and incomplete colonoscopic diagnosis were excluded from the analysis.

**Figure 1:**
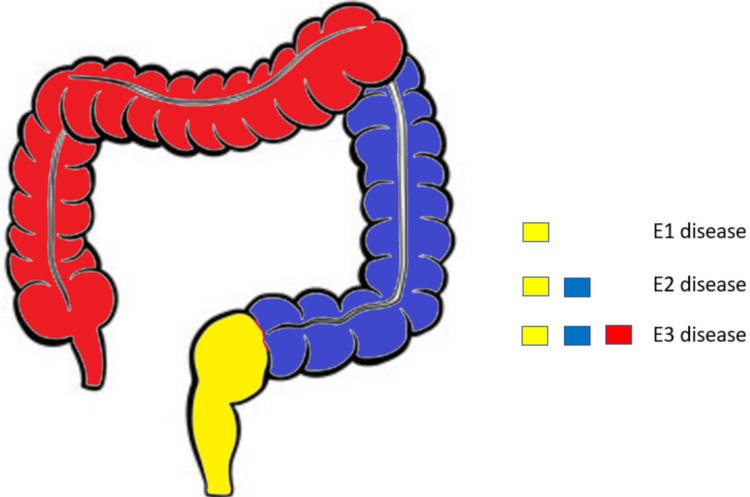
Montreal classification of disease extent in ulcerative colitis

The extent of disease in patients with ulcerative colitis was described according to the Montreal classification (Figure 1) [12].

Data were entered in Microsoft Excel (Microsoft Corporation, Redmond, WA) and analyzed by SPSS 22 software (IBM Corp., Armonk, NY). Continuous data were expressed as mean (SD), median and interquartile range (IQR), and categorical variable as number (%).

## Results

**Figure 2:**
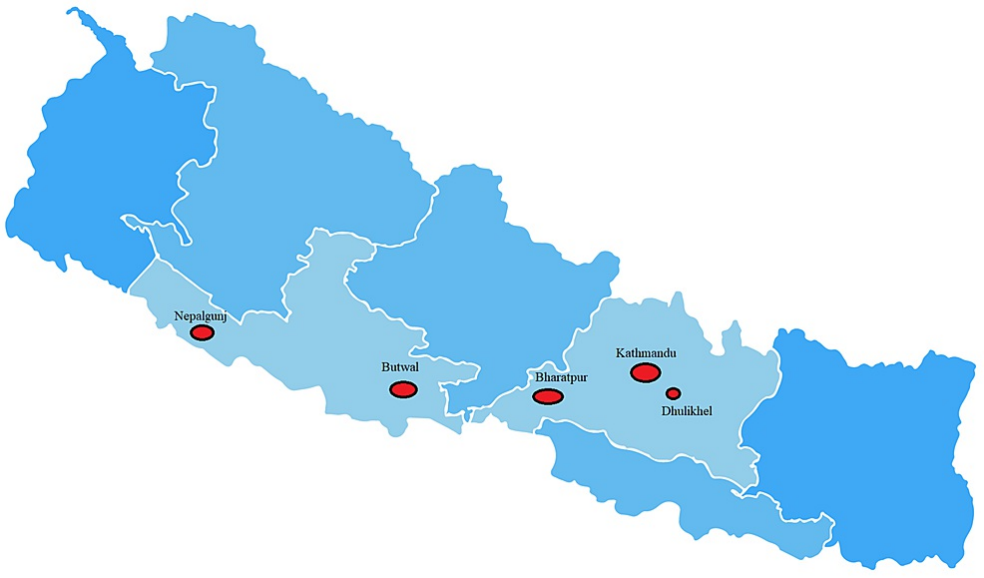
Location of study centers in Nepal

Nine centers, located in various parts of Nepal, participated in the study. One center was from Nepalgunj, one center from Butwal, two centers from Bharatpur, four centers from Kathmandu, and one center from Dhulikhel participated in the study. Data regarding the total number of colonoscopies for two years were obtained only from six centers; one center provided overall colonoscopies data for one year only and the remaining two centers provided only IBD data of two years. UC was identified in 352 patients from 7423 colonoscopies, thus the incidence rate of UC was calculated as 23.7 per 1000 colonoscopies per year. CD was identified in 24 cases out of 7423 colonoscopies, and the incidence rate was calculated as 1.61 per 1000 colonoscopies per year (Figure 2).

**Figure 3:**
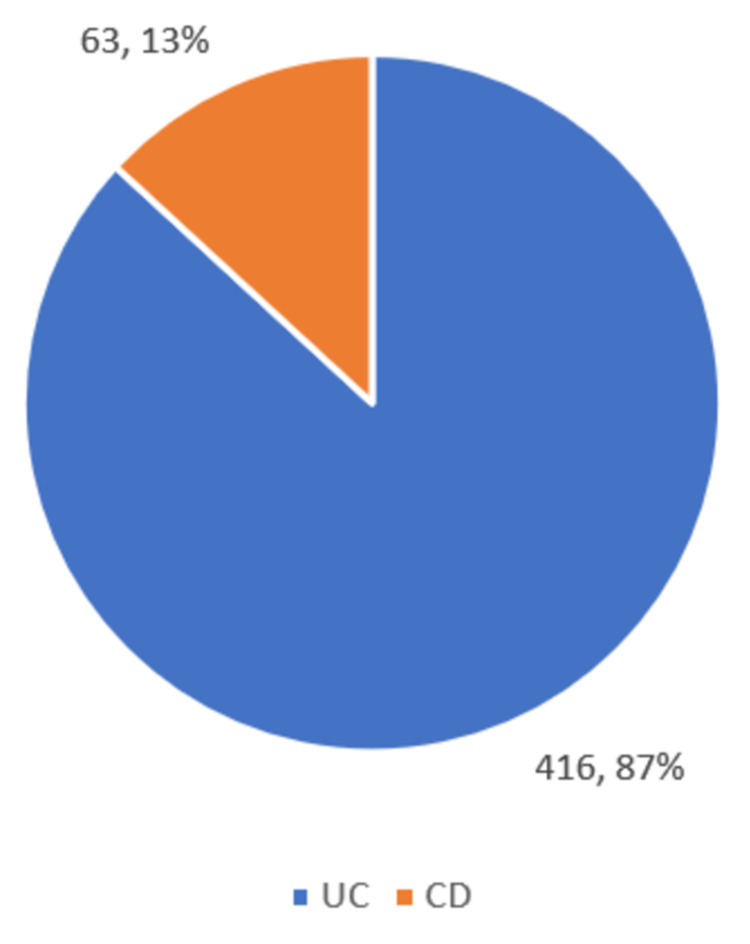
Distribution of patients with IBD in the study IBD: inflammatory bowel diseases; UC: ulcerative colitis; CD: Crohn's disease

A total of 7526 patients were included in this study. The total number of patients with IBD was 479 (6.36%). Ulcerative colitis was the predominant IBD and was seen in 416 patients. Crohn's disease was seen in only 63 patients. The distribution of IBD is shown in Figure 3.

The study population consisted predominantly of males (58.9%). Among patients with UC, 245 (58.8%) were males and 171 (41.1%) were females, whereas among patients with CD, 39 (61.9%) were males and 24 were females (38.0%).

**Figure 4:**
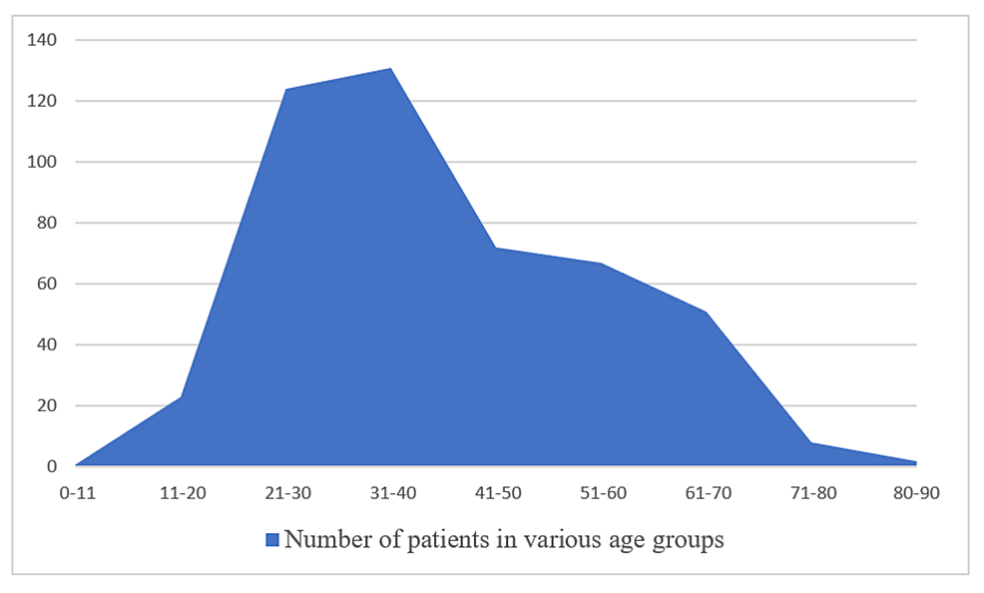
Distribution of patients in various age groups (age in years)

The average age of patients included in our study was 45.8 years The average age of patients with UC in our study was 40.6 years, and the average age of patients with CD was 36.9 years. The distribution of patients in various age groups is shown in Figure 4.

Out of 416 patients with UC, data regarding the location of lesions during colonoscopy could be collected in only 268 patients (Table 1).

**Table 1:** Extent of colonic involvement in patients with ulcerative colitis (UC)

Location of lesions in UC	Number of patients n (%)
E1 (Ulcerative proctitis)	127 (30.52%)
E2 (Left-sided UC)	75 (18.02%)
E3 (Extensive colitis)	66 (15.86%)

## Discussion

Nepal is a lower-middle-income country (LMIC) and thus has an emerging problem of healthcare-related issues, including IBD. In 2020, the majority of the population of Nepal resided in rural areas (81%). Only 61.8% of Nepalese households had access to health facilities within 30 minutes, with a significant urban (85.9%) and rural (59%) discrepancy [13]. Nepal also lacks a national IBD registry. Our study has shown that in Nepal, UC is more prevalent than CD, males are affected more than females by both UC and CD and IBD is more common in the age group of 30 to 40 years. Ours is the largest study to date from Nepal which looked into the age and gender distribution of patients with IBD.

The majority of the centers in our study were located in urban regions. Because Nepal is a hilly country, the rural population does not have easy access to health care services. This may have created a bias in the inclusion of patients in our study. However, studies have also suggested that IBD is predominantly a disease of the urban population [14]. This is also supported by the observation that IBD is increasing along with urbanization in many countries.

UC is predominantly a disease of the colon [15]. In some cases, terminal ileum may be involved and this condition is termed backwash ileitis [16]. CD, however, may involve the entire gastrointestinal tract from mouth to the anus. On colonoscopic examination, UC presents with rectal lesions with continuous involvement. CD presents with skip lesions with frequent involvement of the ileocecal region. In some cases, CD and UC may be difficult to be distinguished clinically, endoscopically, or histologically, which are then classified as indeterminate colitis [3]. An International Working Group has recommended restricting the term IC to resected specimens and to use “IBD unclassified” (IBD-U) for all other cases [3,17]. 

The epidemiology of IBD has not been studied well in Nepal. A single case each of ulcerative colitis and Crohn's disease was reported in 1980 from Nepal, out of 1729 cases of bloody diarrhea [18]. In a large center from eastern Nepal, the first-ever case of Crohn's disease was reported only in 2009 [19]. Various other studies have looked into the demographic profile, incidence, and prevalence of IBD in Nepal. A summary of studies on IBD from Nepal is shown in Table 2.

**Table 2:** Summary of studies on IBD in Nepal IBD: inflammatory bowel diseases; UC: ulcerative colitis; CD: Crohn's disease Sources: [8-11]

Study design	Study period	Patient characteristics	Number of patients with UC	Number of patients with CD	Average age	M: F ratio	Incidence	Prevalence
Retrospective	2009 to 2017	Patients in the surgical unit	19	11	UC: 45.1 years, CD: 55 years	UC=1.3:1, CD=1.7:1	2009 to 2015: 1.8 cases per year, 2015 to 2017: 9.5 cases per year	-
Cross-sectional	2014 to 2015	Patients with UC	60	-	34.6 years	1.06:1	-	-
Cross-sectional	2016 to 2018	Patients with UC	100	-	38.04 years	1.04:1	-	-
Prospective observational	2017 to 2020	Patients with a colonoscopic diagnosis of IBD	60	4	UC: 37 years	UC=1.5:1	-	UC: 21.9%

One of the aims of this study was to identify the age of affliction with IBD in Nepali patients. This would help in directing awareness programs to the target age group. In a study from Ontario, which has among the highest prevalence of IBD in the world, the incidence of IBD has increased between 1999 to 2008 among children and younger population, whereas the incidence is relatively stable among those more than 65 years of age [20]. In a systematic review and meta-analysis from China, which included 2,283 CD and 17,958 UC cases, the majority of CD patients were diagnosed between 17 and 40 years of age but 63.9% of UC cases were diagnosed when the patient was older than 40 years [21]. Unlike in the west, however, most of the studies in Asia do not show a second peak in IBD incidence in the sixth to eighth decades [6]. The average age of diagnosis of UC and CD in our study was between 30 and 40 years of age. There was no second peak of IBD occurrence at an older age in our study.

Immune-mediated diseases are more prevalent among females than males. In IBD, gender-specific differences have been reported for Crohn’s disease (CD), but not ulcerative colitis (UC), although data are conflicting and possibly depend on geographic areas [22]. In our study, a male predominance was seen in both UC (1.4:1) and CD (1.7:1).

We also attempted to classify the disease location in patients with UC based on colonoscopic findings. Although the data was unavailable from a majority of centers regarding this parameter, the majority of our patients with UC had rectal involvement only. This is plausible, as UC is known to start from the rectum and progress proximally in the colon.

There were a few limitations of our study. The data was collected retrospectively and most of the study centers had an urban location. Thus, there might have been selection bias on the nature of patients presenting to the study centers. This also might explain predominantly younger and male patients in our study. The other limitation is the lack of IBD-U as a diagnosis, suggesting we might be overdiagnosing Crohn’s colitis or even lack physician knowledge about the existence of IBD-U. Also, since we only used endoscopic assessment as the modality of IBD diagnosis, we might have missed other diseases with similar endoscopic features.

## Conclusions

IBD in Nepal affects young males in their thirties, which is a working-age group. The incidence of CD and UC is on the rise with increasing urbanization. Lack of awareness regarding symptoms and the non-availability of diagnostic modalities of IBD may result in a delayed diagnosis. The target population has to be made aware of the symptoms, diagnosis, and treatment modalities of IBD with the help of various government and non-government agencies. There is also a need of creating a national IBD registry for Nepal.
